# Neuroendocrine tumor arising from the greater omentum treated with laparoscopic tumor resection: a case report

**DOI:** 10.1186/s40792-021-01217-4

**Published:** 2021-06-01

**Authors:** Taichi Terai, Kenji Nakagawa, Kota Nakamura, Shunsuke Doi, Kohei Morita, Chiho Ohbayashi, Masayuki Sho

**Affiliations:** 1 Department of Surgery, Nara Medical University, 840 Shijo-cho, Kashihara, Nara 634-8522 Japan; 2 Department of Diagnostic Pathology, Nara Medical University, 840 Shijo-cho, Kashihara, Nara 634-8522 Japan

**Keywords:** Omental tumor, Neuroendocrine tumor, Laparoscopic surgery

## Abstract

**Background:**

Primary omental tumors are extremely rare. Herein, we report the first case of a primary omental neuroendocrine tumor (NET).

**Case presentation:**

A 59-year-old woman was referred to our hospital for the treatment of an 18-mm tumor located at the ventral side of the duodenum. No other tumor was detected. The preoperative imaging diagnosis was omental tumor. A laparoscopic tumor resection was performed. Histopathological examination revealed that the tumor consisted of cuboidal cells with eosinophilic, granular cytoplasm showing trabecular or ribbon architecture. No other component was seen. The mitotic count was of 5 per 10 high-power fields. Immunohistochemical staining was positive for chromogranin A, synaptophysin, and CD56. Her Ki-67 index was 5%. These results led to the diagnosis of grade 2 omental NET. The patient was discharged on the 3rd postoperative day without any complications and did not develop any recurrence for 3 years.

**Conclusions:**

We encountered a very rare case of omental NET. Complete resection is recommended with minimally invasive surgery for the diagnosis of NET.

**Supplementary Information:**

The online version contains supplementary material available at 10.1186/s40792-021-01217-4.

## Background

Neuroendocrine neoplasms (NENs) are most commonly located in the gastrointestinal tract (approximately 68% of patients), followed by the respiratory tract (25%), whereas other locations are extremely rare [1]. The neuroendocrine cells are located throughout the body, such as the skin, mucosal membranes, and all solid organs. Therefore, NENs can originate from almost every location [2]. However, an omental NEN has never been reported previously. Herein, based on our literature review, this is the first report on omental neuroendocrine tumor (NET) treated with laparoscopic tumor resection worldwide.

## Case presentation

**Fig. 1:**
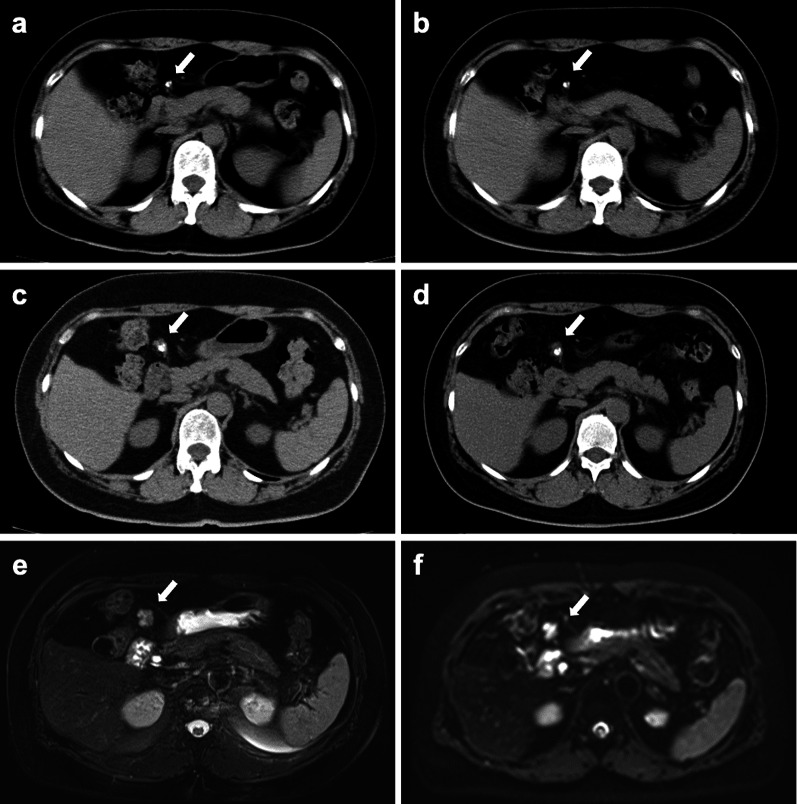
Computed tomography and magnetic resonance images. **a**–**d** Computed tomography revealed a gradually growing tumor. **e** T2-weighted magnetic resonance image showed a faint high-signal-intensity tumor. **f** Diffusion-weighted magnetic resonance image showed that tumor was high-signal intensity

A 59-year-old woman was referred to our hospital for the treatment of a growing tumor detected by computed tomography (CT) with calcification on the ventral side of the duodenum. Six years before the presentation, a nodule with calcification that had grown from 6 mm to 7.5, 15, and 18 mm at 14-, 56-, and 68-month follow-up, respectively, in the CT (Fig. 1a–d). Then she was referred our department for further evaluation and treatment. She had no chief symptoms. She had a history of chronic gastritis, gastroesophageal reflux disease. Her family history was unremarkable. On physical examination, she had a soft and flat abdomen without palpable masses. Laboratory tests, including tumor markers, showed no abnormalities. The tumor showed a faint high-signal intensity on T2-weighted magnetic resonance imaging (MRI) (Fig. 1e) and high-signal intensity on diffusion-weighted MRI (Fig. 1f). No other tumor was detected on endoscopy. The diagnosis was an omental tumor. The tumor was gradually growing and suspected as a malignant lesion. Therefore, laparoscopic surgery was performed.

**Fig. 2:**
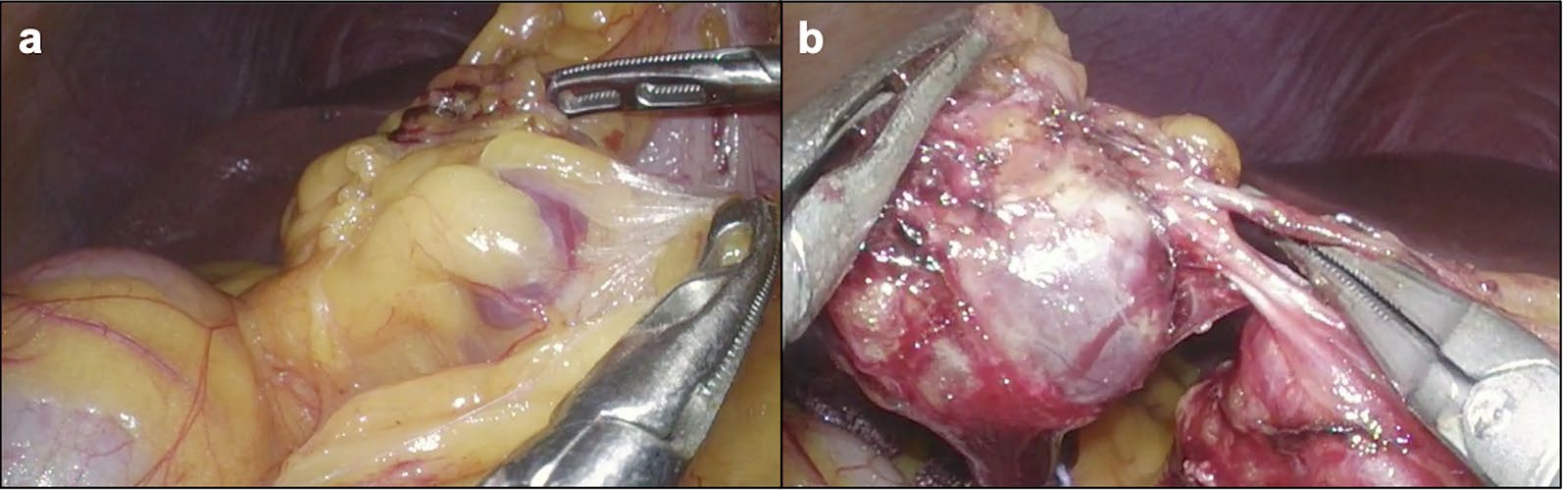
Intraoperative findings. **a** The tumor was identified at the omentum. **b** The tumor was attached to a branch of the RGV

The patient was placed in the supine position under general anesthesia. Five trocars were introduced, similar to laparoscopic distal gastrectomy. The tumor was easily identified at the omentum near the right gastroepiploic vein (RGV) (Fig. 2). Sharp dissection was accomplished using a Sonosurg™ ultrasonic dissection device (Olympus, Tokyo, Japan). The RGV branch was divided after clipping (Additional file 1: Video S1). The total operative time was 99 min, and the intraoperative blood loss was low.

**Fig. 3:**
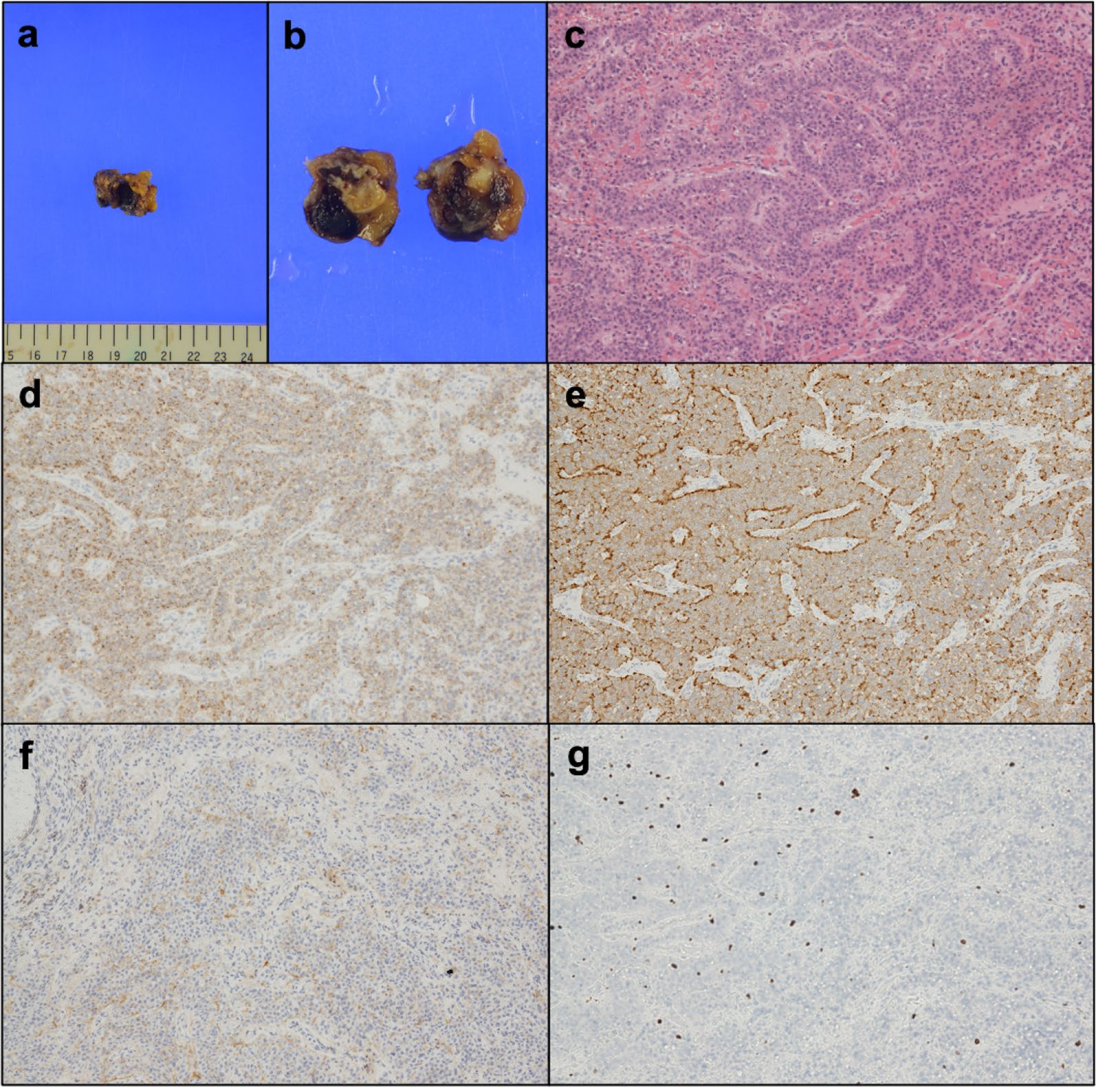
Macroscopic and histological findings. **a**, **b** Resected specimen of omentum having a brown ovoid mass, measuring 20 mm. **c**–**g** Microscopic findings. Trabecular or ribbon growth of cuboidal cells with microvessels (**c** HE stain, × 10). Tumor cells were positive for chromogranin A (**d** × 10), synaptophysin (**e** × 10), CD56 (**f** × 10). Ki-67 index was 5% (**g** × 10)

Macroscopically, tumor showed well-circumscribed brown to black ovoid mass measuring 20 mm (Fig. 3a, b). Histologically, tumor consisted of cuboidal cells with eosinophilic, granular cytoplasm showing trabecular or ribbon architecture. Nucleus of tumor cells had coarsely granular chromatin and mitotic figure (5 cells/10 HPF). No other component, including heterotopic tissue such as aberrant pancreas or lymph node, was seen. Immunohistochemically, tumor cells were positive for chromogranin A, synaptophysin, and CD56, whereas negative for CK7, CK20, estrogen receptor, and progesterone receptor. The Ki-67 index was 5% (Fig. 3c–g). Based on these findings, the tumor was diagnosed as a grade 2 NET, according to the World Health Organization 2019 classification.

The postoperative course was uneventful, and the patient was discharged on postoperative day 3. She developed no signs of recurrence by CT/MRI follow-up at 42 months. No other primary suspected lesion was detected on endoscopic examination. Therefore, we diagnosed the omental NET as the primary lesion.

## Discussion

NENs are relatively rare; they mainly develop in the gastrointestinal tract, accounting for < 2% of all primary gastrointestinal tumors [3]. Primary NENs in the gastrointestinal tract occur most frequently in the rectum, small intestine, pancreas, stomach, colon, duodenum, and appendix. Among them, omental NENs are extremely rare. Based on our literature review, it has not been reported before. The rare incidence of NENs in the omentum can be explained absence of neuroendocrine cells in the said location. In the gallbladder, a similarly rare origin of NENs, undifferentiated gallbladder stem cells are considered to separate into neuroendocrine cells [4]. In omental NENs, multipotent stem cells might be involved in some way.

The omentum could be a metastatic site from the stomach, gallbladder, pancreas, large bowel, and ovaries. Therefore, it is important to examine the whole body to find any possibility of another lesion. In some reports, In-111 octreotide scintigraphy is considered useful for whole-body screening with sensitivity of 80–90% [5, 6]. In our case, the malignant tumor cannot be diagnosed as a NET preoperatively and octreotide scan was not performed. Instead, a whole-body CT, MRI and endoscope were performed for screening. At present, no other growing tumors have developed after 42 months of careful follow-up.

Primary solid tumors of the greater omentum are rare and sporadic. Their etiological factors are still unknown. In a review of the literature, Fagkrezos et al. reported 54 cases of extra-gastrointestinal stromal tumors [7], Barel et al. reported 27 cases of leiomyosarcoma [8], and Hashimoto et al. reported 19 cases of liposarcoma of the greater omentum [9]. According to these reports, the preoperative diagnosis of primary omental tumor is difficult because of its rarity, and thus, the diagnosis is usually made postoperatively. Accurate diagnosis is only obtained through the histopathological examination of the tumor [10]. Preoperative transabdominal biopsy may be considered for the diagnosis of omental tumors; however, it may cause organ injury and peritoneal dissemination. Therefore, laparoscopic tumor resection is reasonable and safe for the diagnosis and treatment of NENs.

## Conclusions

We encountered a very rare case of omental NET. Complete resection by minimally invasive surgery is recommended for diagnosis because of the difficulty of preoperative diagnosis.

## Supplementary Information

**Additional file 1.** The tumor was easily identified at the omentum near the RGV.

## Data Availability

Not applicable.

## Abbreviations

NEN: Neuroendocrine neoplasm
NET: Neuroendocrine tumor
CT: Computed tomography
MRI: Magnetic resonance image
RGV: Right gastroepiploic vein
